# Concerted evolution and unorthodox recombination of human subtelomeres

**DOI:** 10.64898/2026.07.10.737660

**Published:** 2026-07-31

**Authors:** Andrea Guarracino, Angela Gyamfi, Human Pangenome Reference Consortium, Erik Garrison

## Abstract

Human subtelomeres contain duplicated sequence that is shared among the ends of non-homologous chromosomes and provides a substrate for ectopic exchange. However, incomplete reference assemblies and chromosome-by-chromosome analyses have prevented a population-scale view of the extent and organization of subtelomeric exchange. Here we apply a reference-free pangenome approach to 465 near-complete human assemblies, comparing every chromosome end against every other, and find that high-identity pseudo-homolog regions occur on 41 of 48 chromosome arms. These regions group into sequence communities, sets of chromosome ends that share duplicated sequence. Across these communities, copy-number enrichment is concentrated in gene modules associated with chromosome dynamics, intracellular organization and germ-cell development. Human chromosome-contact maps show preferential proximity between subtelomeres with similar sequences, persisting even in adjacent flanks that lack the shared sequence used to define each pair. Mouse contact maps show the same preference, strongest in meiosis at the zygotene bouquet, when telomeres cluster at the nuclear envelope. In a three-generation telomere-to-telomere pedigree, whole-genome comparison identifies putative recombination between subtelomeric regions on non-homologous chromosomes from the same sequence communities, while recovering the obligate X-Y crossover in PAR1, the pseudoautosomal region shared by the X and Y chromosomes, during male meiosis. These results generalize known subtelomeric exchange systems into a near-ubiquitous architecture and support recurrent ectopic exchange as a genome-wide force in the concerted evolution of human chromosome ends.

## Full Text Availability

The license terms selected by the author(s) for this preprint version do not permit archiving in PMC. The full text is available from the preprint server.

